# β2-Adrenergic Receptor Enhances the Alternatively Activated Macrophages and Promotes Biliary Injuries Caused by Helminth Infection

**DOI:** 10.3389/fimmu.2021.754208

**Published:** 2021-10-18

**Authors:** Stephane Koda, Beibei Zhang, Qian-Yang Zhou, Na Xu, Jing Li, Ji-Xin Liu, Man Liu, Zi-Yan Lv, Jian-Ling Wang, Yanbiao Shi, Sijia Gao, Qian Yu, Xiang-Yang Li, Yin-Hai Xu, Jia-Xu Chen, B. Oneill Telakeng Tekengne, Gabriel K. Adzika, Ren-Xian Tang, Hong Sun, Kui-Yang Zheng, Chao Yan

**Affiliations:** ^1^ Jiangsu Key Laboratory of Immunity and Metabolism, Department of Pathogenic Biology and Immunology, Xuzhou Laboratory of Infection and Immunity, Xuzhou Medical University, Xuzhou, China; ^2^ National Demonstration Center for Experimental Basic Medical Science Education, Xuzhou Medical University, Xuzhou, China; ^3^ Department of Dermatology, The Second Affiliated Hospital of Nanjing Medical University, Nanjing, China; ^4^ Department of Laboratory Medicine, The Affiliated Hospital of Xuzhou Medical University, Xuzhou, China; ^5^ National Institute of Parasitic Diseases, Chinese Center for Disease Control and Prevention, Key Laboratory of Parasite and Vector Biology, Ministry of Health, World Health Organization (WHO) Collaborating Center of Malaria, Schistosomiasis, and Filariasis, Shanghai, China; ^6^ Department of Clinical Medicine, Xuzhou Medical University, Xuzhou, China; ^7^ Department of Physiology, Xuzhou Medical University, Xuzhou, China

**Keywords:** beta 2 adrenergic receptor, macrophages, liver fibrosis, *Clonorchis sinensis*, ERK/mTORC1 signaling

## Abstract

The autonomic nervous system has been studied for its involvement in the control of macrophages; however, the mechanisms underlying the interaction between the adrenergic receptors and alternatively activated macrophages (M2) remain obscure. Using FVB wild-type and beta 2 adrenergic receptors knockout, we found that β2-AR deficiency alleviates hepatobiliary damage in mice infected with *C. sinensis*. Moreover, β2-AR-deficient mice decrease the activation and infiltration of M2 macrophages and decrease the production of type 2 cytokines, which are associated with a significant decrease in liver fibrosis in infected mice. Our *in vitro* results on bone marrow–derived macrophages revealed that macrophages from *Adrb2^−/−^* mice significantly decrease M2 markers and the phosphorylation of ERK/mTORC1 induced by IL-4 compared to that observed in M2 macrophages from *Adrb2^+/+^*. This study provides a better understanding of the mechanisms by which the β2-AR enhances type 2 immune response through the ERK/mTORC1 signaling pathway in macrophages and their role in liver fibrosis.

## Introduction

Macrophages are a key element in the initiation of the immune response (1). Macrophages can modify their phenotype and the type of response according to different types of stimuli. Thus, macrophages exposed to stimuli arising from the type 1 immune response such as interferon-gamma (IFN-γ) or lipopolysaccharide (LPS) differentiate into classically activated macrophages (M1) characterized by the high production of pro-inflammatory cytokines (2, 3), whereas macrophages exposed to stimuli such as interleukin (IL)-4 and IL-13 (type 2 immune cytokine) differentiate into alternatively activated macrophages (AAMs) or M2 with the high expression of Arginase-1, Fizz-1, chitinase-like protein 3 (Chil3)[Ym1], scavenger receptors such as CD163, and mannose receptors (CD206), as well as the production of profibrotic cytokines (3–6).

The activation of macrophages is a process that is highly regulated. The involvement of the autonomic nervous system in the control of macrophages has been studied in recent years, but mechanisms underlying the interaction between the nervous system and activation of macrophages in the context of type 2 immune response remain obscure. Catecholamines produced by neurons of the sympathetic nervous system and those released into the bloodstream by the pituitary gland activate different effectors located on the membranes of macrophages and binding with adrenergic receptors allowing their control (7). Beta 2 adrenergic receptor (β2-AR) is such kind of receptor that can control the activation of macrophages (8, 9). In macrophages, many data have shown that activation of β2-AR has immunosuppressive properties by reducing the pro-inflammatory response *via* the canonical cAMP/PKA pathway (10). In addition, Grailer et al. (2) showed that the activation of β2-AR by norepinephrine not only reduces the production of pro-inflammatory cytokines such as TNF-α, IL-12, and CXCL chemokines but also increases the production of IL-10 in the condition of LPS-induced acute lung injury. However, the regulatory mechanism by which β2-AR orchestrates AAMs in the context of type 2 immune response has so far not been investigated.

*Clonorchis sinensis* (*C. sinensis*) is a helminth that dwells in the bile ducts for a long time (almost 20~30 years if not treated) (11). Infection with *C. sinensis* can lead to cholestasis, cholangitis, and biliary fibrosis, which finally cause liver cirrhosis and possibly cholangiocarcinoma (12, 13). During infection with *C. sinensis*, the contact of the parasite with its host triggers the activation of immune cells such as macrophages, eosinophils, mast cells, basophils, Th2 cells, and group 2 innate lymphoid cells (ILCs) (14). The resulting production of type 2 cytokines (IL-4 and IL-13) induces the differentiation of macrophages into AAMs (M2), which then begins the repair process and the restoration of the homeostasis (15, 16). However, the signaling by which β2-AR orchestrates AAMs and promotes biliary fibrosis caused by helminth infection is still unknown.

In the present study, we used FVB wild-type mice (*Adrb2^+/+^*) and β2-AR knockout mice (*Adrb2* ^−^ *^/^* ^−^) with FVB background because of their susceptibility to helminth infection and their ability to elicit a predominant type 2 immune response. This study allowed us to explore and understand the mechanism by which β2-AR regulates M2 macrophages and promotes liver fibrosis induced by *C. sinensis* infection. In our present study, we found that β2-AR can regulate the response of alternatively activated macrophages during liver fibrosis induced by *C. sinensis* infection. Moreover, the β2-AR-orchestrated activation of M2 is mediated by the mTORC1. This study provides a better understanding of the mechanisms by which the β2-AR enhances type 2 immune response through the ERK/mTORC1 signaling pathway in macrophages and their role in liver fibrosis.

## Material and Methods

### Ethics Statement

All animals’ experimental procedures were strictly performed according to the Guidelines for Animal Experiments of Xuzhou Medical University and the National Guide for the Care and Use of Laboratory Animals. The study protocol was reviewed and approved by the Committee on Ethics of Animal Experiments, Xuzhou Medical University (201701w007).

### Bone Marrow–Derived Macrophages Culture

Bone marrow (BM) cells were flushed from femur and tibia of *Adrb2^+/+^* and *Adrb2^−/−^* mice and cultured using DMEM supplemented with 20% (v/v) FBS, 1% (v/v) P/S, 20 ng/ml mouse recombined murine M-CSF (Proprotech, USA) for 7 days (17). The purity of BM-macrophage cultures was confirmed by FACS using CD11b (1:100, BioLegend, San Diego, CA, USA) and F4/80 (1:100, BioLegend, San Diego, CA, USA) antibodies. Seven days later, BM-derived macrophages were cultured in a DMEM medium (Invitrogen, Carlsbad, CA, USA) for 24 h to remove the residual M-CSF. M0 macrophages (non-differentiated macrophages) were then stimulated with recombinant IL-4 (rIL4) (20 ng/ml, Proprotech, USA) with or without mTORC1 inhibitor rapamycin (100 ng/ml, MCE, USA) for 24 h. After that, the rIL-4 was removed and the cells were washed to remove the remaining rIL-4 and collected for further analysis. For measuring IL-4, the cells were washed and the DMEM medium (Invitrogen, Carlsbad, CA, USA) was added for another 24 h.

### Preparation of *C. sinensis* Metacercariae

*C. sinensis* metacercariae were obtained as previously described (18, 19). Briefly, the meat from *Pseudorasbora parva* (a kind of freshwater fish) containing *C. sinensis* metacercariae was minced and digested with artificial gastric juice, a solution of 0.7% pepsin A and 0.1% HCl, at 37°C in a shaking water bath for 12 h. The digested mixture was filtered through a sieve with a mesh size of 100 μm. Then the pellet was sedimented in a phosphate-buffered solution (PBS) in a sedimentation jar until the supernatant was clear. *C. sinensis* metacercariae were identified, collected under a dissecting microscope, and stored in PBS at 4°C until use.

### Animal Experiments

Males FVB wild-type mice (*Adrb2^+/+^*) were purchased from the Beijing Vital River Laboratory Animal Technology Co. Ltd. β2-AR knockout mice (*Adrb2^−/−^*) were kindly gifted by Prof. Hong Sun from the Department of Physiology, Xuzhou Medical University. All mice were bred and maintained at the Animal Center of Xuzhou Medical University (Xuzhou, Jiangsu, China). All the mice were group-housed in a specific pathogen-free condition with the temperature-control 24°C with a 12 h dark/light cycle and permitted free access to food and water at the Animal Center of Xuzhou Medical University (Xuzhou, Jiangsu, China). The experimental procedure was carried out on 6–10-week-old mice as follows: two main groups including FVB wild-type (*Adrb2^+/+^*) and β2-AR knockout mice (*Adrb2^−/−^*) were each divided into the control group and the infected group. In each mouse of the infected group, 45 metacercariae were intragastrically administered, and the irrigating solution was observed under the microscope to ensure that all metacercariae were completely infused; the mice of the non-infected group were given the same volume of normal saline. Four weeks later, the metacercariae were able to develop into adult worms and release eggs, with the development of liver fibrosis (20). On the 28th day of infection in mice, serum and liver tissue were collected for further study.

### Liver Function Test

The activities of alanine aminotransferase (ALT), aspartate aminotransferase (AST), total bilirubin (TBIL), alkaline phosphatase (ALP), total bile acid (TBA), and Cellular cholesteryl ester (CHE) in plasma (Department of Laboratory Medicine, Affiliated Hospital of Xuzhou Medical University, China) were assayed to indicate liver function in infected and uninfected mice.

### Histology, Immunohistochemistry, and Immunofluorescence Staining

To evaluate the histopathological changes in *C. sinensis*–infected mice and their capacity to induced cholangiopathy, the liver tissue was serially sectioned at 4 μm for H&E staining according to the manufacturer’s instructions (Jiangsu Beyotime Biotechnology Research Institute, China). After sealing the slides with neutral adhesive, the pathological changes of stained histological sections were observed by microscope (Olympus, Japan). Tissue sections were stained with H&E; and hyperplasia, epithelial damage, infiltration of inflammatory cells, and histological scoring were determined based on the histological findings.

For IHC and IF staining, serial sections of embedded tissue from each mouse were used for the staining of cytokeratin 19 (CK-19), CD206, CD68, Arginase-1, and β2-AR. The liver tissue was deparaffinized, hydrated, and heated in citric acid buffer at 95°C for 15 min and then blocked with 5% BSA for 30 min. The slides were then incubated overnight with primary Anti-Cytokeratin 19 (1:200, ab133496, Abcam, Cambridge, USA), Anti-CD206 (1:400, CST 24595 Danvers, MA, USA), Anti-CD68 (1:200, ab201973, Abcam, Cambridge, USA), Anti-Arginase-1 (1:400, 16001-1-AP, Proteintech, USA), and anti-β2-AR antibody (1:200, ET1703-04, Huabio, China). After washing with PBS, DAB (1:200, ZSGB–BIO, Beijing, China) as an enzyme-substrate was added. Five high-power fields (×400 magnifications, Olympus, Japan) were randomly selected from each mouse-staining section. CK19 and β2-AR-positive expressions integrated optical density IOD (Integral optical density) was calculated by Image-Pro Plus 6.0 software. A higher IOD value indicates a stronger positive expression.

### Masson’s Staining

Four percent paraformaldehyde was used to fix the liver tissue from each strain mice, and then the liver was embedded in paraffin. Four-μm-thick sections were prepared and stained with Masson’s trichrome, according to the manufacturer’s instructions (Jiancheng, Nanjing, Jiangsu, China). The sections were observed under the microscope and digitized using an imaging system (Olympus, Japan). Five high-power visual fields (200× and 100× magnifications, Olympus, Japan) were randomly selected from the staining sections of each mouse, and Image Pro Plus6.0 software was used to calculate the Integral optical density (IOD) of fibrous tissue. Higher IOD value means stronger positive expression

### Measurement of Hepatic Hydroxyproline Content

To quantify liver fibrosis, hepatic hydroxyproline content was determined according to the instructions of the manufacturer (Jiancheng Institute of Biotechnology, Nanjing, China) according to the manufacturer’s recommendations. Hydroxyproline content was measured at 570 nm using a hydroxyproline standard curve.

### RNA Isolation and Reverse Transcription

Total RNA was extracted from liver tissue and bone marrow–derived macrophage (BMDM) cells by using TRIzol reagent according to the manufacturer’s instructions (Invitrogen, Carlsbad, CA, USA). The RNA was reverse transcribed to complementary DNA. Quantitative real-time PCR was performed using SYBR green real-time PCR master mix according to the manufacturer’s instructions (Roche Applied Science, Mannheim, Germany). Relative quantification of each gene expression was calculated in terms of the comparative cycle threshold (Ct) normalized by beta-actin (Shanghai General Biotech Co. China).

### Enzyme-Linked Immunosorbent Assay

Serum and mouse liver homogenate from each mouse were immediately subjected to evaluate the concentrations of IL-4, IL-6, IL-10, IL-13, TNF-α by a commercial ELISA Kit with Plates (Thermo Scientific, USA). All procedures were performed according to the instructions provided by the kit. Concentrations of cytokines in the sera were calculated using standard curves as references.

### Western Blotting

Total protein was extracted from liver tissue and BMDM cells by using a homogenizer and analyzed with a bicinchoninic acid protein concentration assay kit (Beyotime Biotech, Beijing, China). Anti-phospho-PKA (p-PKA, 1:1000, SC54-04, Huabio, China), anti-phospho-PI3K (p-PI3K, 1:1000, AP0854, ABclonal, Cummings Park, USA), anti-phospho-ERK1/2 (p-ERK1/2, 1:1000, AP0490, Bioworld, Bloomington, USA), anti-phospho mTOR (p-mTOR, 1:1000, AP0115, Abcam, Cummings Park, USA), anti-mTOR (1:1000, ET1608-5, Huabio, China), anti-phospho-AKTs473 (p-AKTs473, 1:1000, BS4006, Bioworld, Bloomington, USA), anti-AKT (1:1000, ET1609-47, Huabio, China) anti-phospho-Stat6 (p-Stat6, 1:1000, Ab263947, Abcam, Cambridge, USA), anti-b2-AR (1:1000, ET1703-04, Huabio, China), anti-alpha SMA (1:1000, 14395-1-AP, Proteintech, USA), anti-beta-actin (1:1000, 66009-1-Ig, Proteintech, USA), and anti-GAPDH (1:1000, AC033, ABclonal, Cummings Park, USA) were used as primary antibody after separation by electrophoresis in 7.5 and 10% SDS-PAGE with a Bio-Rad electrophoresis system (Hercules, CA, USA). The secondary antibodies (anti-rabbit IgG, 1:5000, HRP-S0001, Affinity Biosciences, Cincinnati, USA and anti-mouse IgG, 1:5000, 7076S, CA, USA) were incubated for 2 h at room temperature. The membrane containing antibody-protein complexes was visualized with an enhanced chemiluminescence detection system on radiographs film (Bio-rad, Hercules, CA, USA). The bands were scanned and analyzed by the software Quantity ONE (Bio-rad, Hercules, CA, USA). The expression of the protein in each sample was normalized by GAPDH or beta-actin (Santa Cruz Biotechnology, CA, USA).

### Flow Cytometry

The cell suspensions were blocked with an Fc blocker (Anti-mouse CD16/32, 101319, Biolegend, USA) before incubation with surface marker antibodies. The following antibodies were used to stain cell surface markers: Briliant Violet 510 anti-mouse/human CD11b (101236, Biolegend, USA), CD206 Alexa 647 MR5D3 (565250, Biolegend, USA), and PerCP/Cy5.5 anti-mouse F4/80 (123127, Biolegend, USA). For the characterization of M2 macrophages, CD11b and F4/80 were used to define the population of macrophages. After that, CD206 was used to characterize the population of M2 macrophages in the total population of macrophages. The results were analyzed using FlowJo software.

### Statistical Analysis

All the data were expressed as mean ± s.e.m. The data were analyzed by SPSS 23.0 software (SPSS Inc, Chicago, IL, USA). The student *t*-test was used for comparison between the two groups. One-way ANOVA with Tukey’s *post hoc* test was used for comparison for more than two groups. Statistical significance was ranked *P<0.05, **P<0.01, and ***P<0.001.

## Results

### β2-AR Deficiency Alleviates Hepatobiliary Damage in Mice Infected With *C. sinensis*

**Figure 1:**
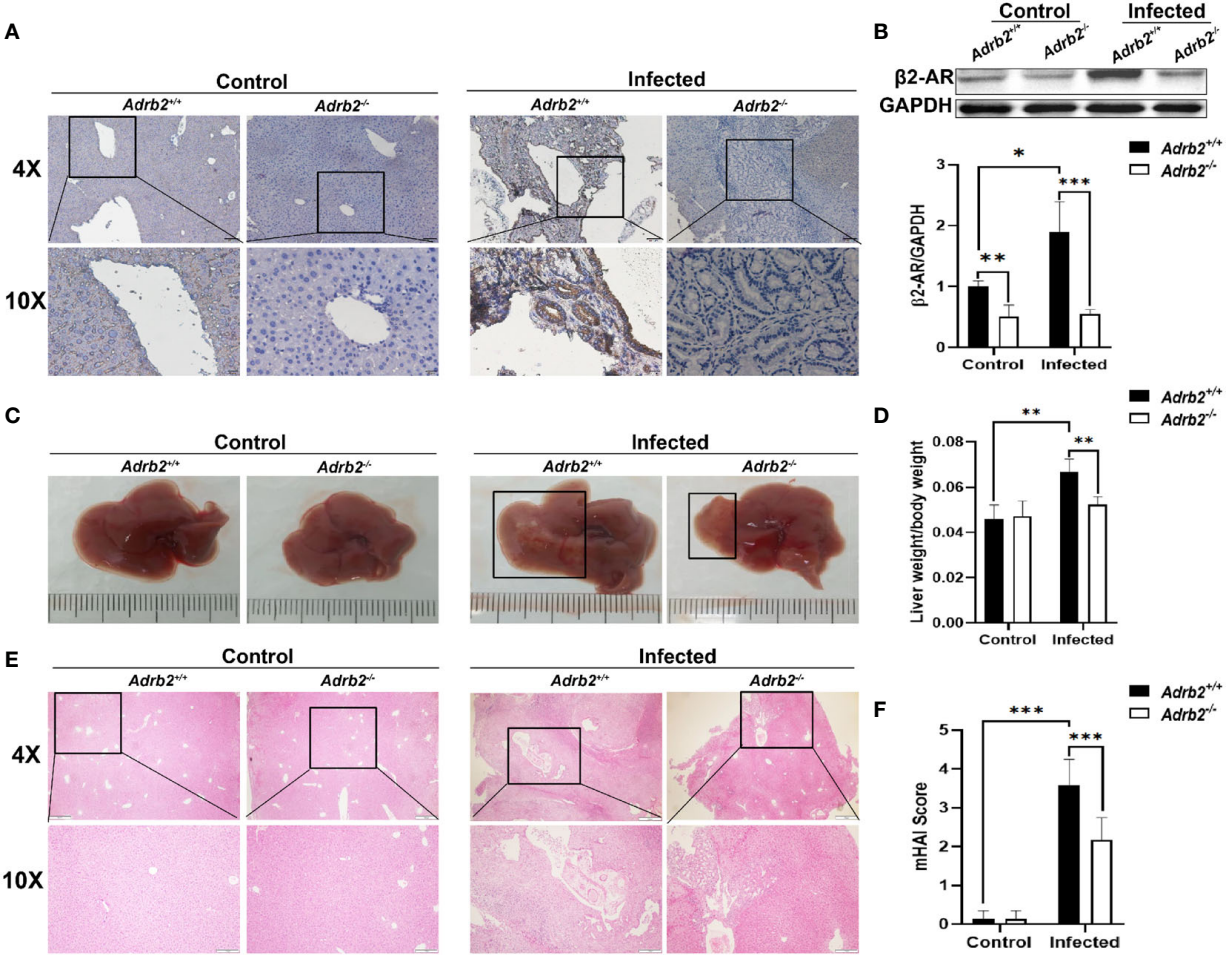
β2-AR deficiency alleviates hepatobiliary damage in mice infected with *Clonorchis sinensis*. The experimental setup of the *in vivo* model used n = 4 mice in the control group and n=6 mice in the infected group. The mice were divided into control *Adrb2^+/^* ^+^, *Adrb2^−/−^* and infected *Adrb2^+/^* ^+^, *Adrb2^−/−^*. **(A)** The Immunohistochemistry analysis of the expression of β2-AR in the liver of control and *C. sinensis* infected mice. The expression of β2-AR significantly increases in infected *Adrb2^+/^* ^+^ than control *Adrb2^+/^* ^+^; however, there is a low or no expression of β2-AR respectively in infected *Adrb2^−/−^* and control *Adrb2^−/−^* mice. **(B)** Western blot results confirm the result of the expression of β2-AR shown by IHC. The expression of β2-AR significantly increases in infected *Adrb2^+/^* ^+^ compared to the other groups. **(C)** The pictures of the liver of control and infected mice. The images of liver injury in infected *Adrb2^+/^* ^+^ is much more severe than that observed in infected *Adrb2^−/−^*. The liver injury in infected *Adrb2^−/−^* is limited to a small part of the liver. **(D)** The liver weight/body weight ratio increases in infected *Adrb2^+/^* ^+^ compared to the others groups. **(E, F)** The Hematoxylin and Eosin analysis of the liver sections revealed that the damage in infected *Adrb2^−/−^* is limited to a small part of the liver with limited necrosis of liver cells, while the liver of infected *Adrb2^+/^* ^+^ shows severe liver injury characterized by the necrosis of tissues in hepatocytes and around the bile duct. Compared with indicated groups, *P < 0.05, **P < 0.01, ***P < 0.001.

Previous studies have shown that beta 2 adrenergic receptors control several functions such as cell proliferation, apoptosis, and immune response (21, 22). In the liver, several types of cells expressed β2-AR (23). In this study, our results showed that the β2-AR are highly expressed in the liver of *Adrb2^+/^* ^+^ (wild-type) mice after *C. sinensis* infection (**Figures 1A, B**). Moreover, there was almost no expression of β2-AR in the liver of *Adrb2^−/−^* regardless of infection or Non-infection in β2-AR-deficient mice (**Figures 1A, B**).

**Figure 2:**
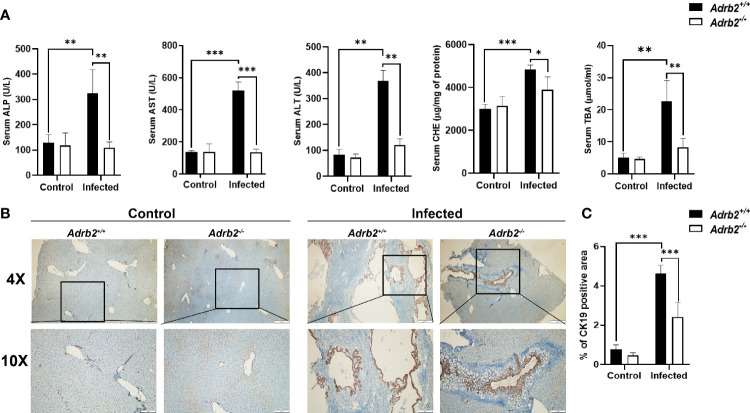
β2-AR deficiency alleviates liver function and cholangiocytes proliferation in mice infected with *Clonorchis sinensis*. The experimental setup of the *in vivo* model used n = 4 mice in the control group and n=6 mice in the infected group. The mice were divided into control *Adrb2^+/^* ^+^, *Adrb2^−/−^* and infected *Adrb2^+/^* ^+^, *Adrb2^−/−^* mice. **(A)** Serum analysis of AST, ALT, ALP, TBA, and CHE showed that the liver function significantly increases in infected *Adrb2^+/^* ^+^, while in deficient mice after *C. sinensis* infection, the liver function significantly decreases almost to the level of normal mice. **(B, C)** The expression of BECs proliferation marker CK19 increases in infected mice compared to that observed in control mice. However, the increase in CK19 in infected *Adrb2^+/^* ^+^ mice significantly increases compared to infected *Adrb2^−/−^* mice. Compared with indicated groups, *P < 0.05, **P < 0.01, ***P < 0.001.

Next, the role of β2-AR in hepatobiliary damage in mice caused by infection with *C. sinensis* was determined. The liver weight/body weight ratio increases significantly in the infected *Adrb2^+/+^* group compared with the non-infected *Adrb2^+/+^* group. However, it decreased in infected *Adrb2^−/−^* mice, compared with the infected *Adrb2^+/+^* mice (**Figures 1C, D**, *P*<0.01). As shown by HE staining in **Figures 1E, F**, the liver structure showed more damages in the infected *Adrb2^+/+^* mice, compared with the non-infected *Adrb2^+/+^* mice, whereas the *Adrb2^−/−^* mice, after *C. sinensis* infection, showed alleviation of liver damages compared to the infected *Adrb2^+/+^* mice. Similarly, observation of the cross-section of the bile duct shows dilatation of the bile duct in infected *Adrb2^+/+^* mice compared to the control group. Also, the dilatation of the bile duct was more evident in the infected *Adrb2^+/+^* than in the infected *Adrb2^−/−^* mice (**Supplementary S1A**). Also, in the infected *Adrb2^+/+^* mice, the liver and biliary damages were significantly increased compared to the control *Adrb2^+/+^* mice as indicated by the measurements of ALP (*P*<0.01), AST (*P*<0.001), ALT (*P*<0.01), CHE (*P*<0.001), and TBA (*P*<0.01), confirming the liver damage caused by *C. sinensis* infection (**Figure 2A**). However, the infected *Adrb2^−/−^* mice significantly decrease the production of ALP (*P*<0.01), AST (*P*<0.001), ALT (*P*<0.01), CHE (*P*<0.05), and TBA (*P*<0.01), compared with the *C. sinensis*–infected wild-type mice. In general, cholangiocytes proliferate in response to an injury, which can act as an indicator of biliary injuries. As shown in **Figures 2B, C**, the infected mice showed a significant increase in the proliferation of cholangiocytes compared to the non-infected control group where the proliferation of cholangiocytes was limited. Also, compared with *Adrb2^+/+^* mice infected with *C. sinensis*, the proliferation of cholangiocyte reduced obviously in *Adrb2^−/−^* mice with infection, as indicated by IHC staining of CK19 (**Figures 2B, C**, *P*<0.001).

### *Adrb2* Deficiency Abates Liver Fibrosis Caused by Infection With *C. sinensis*

**Figure 3:**
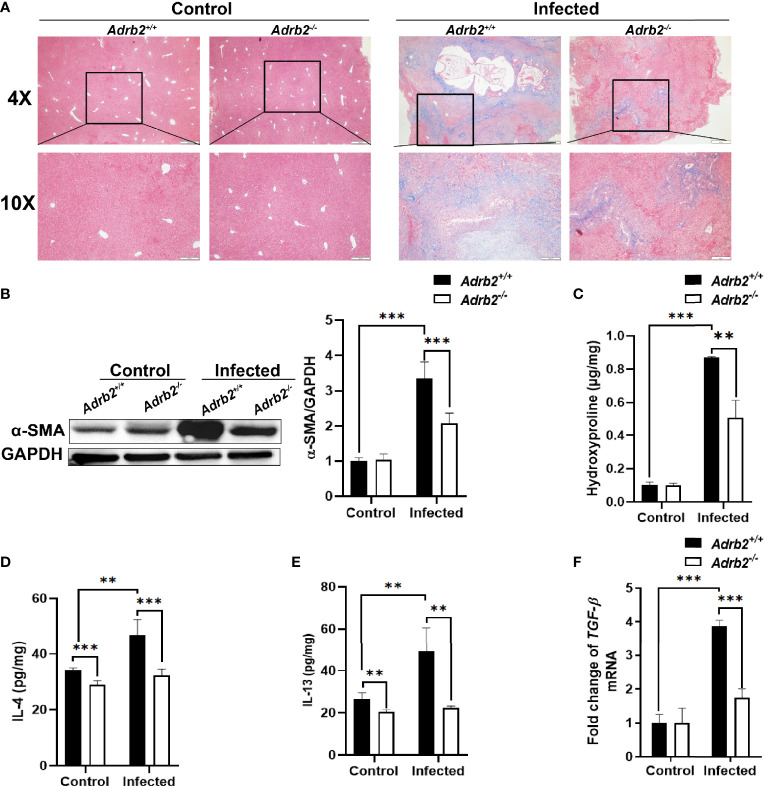
The knocking out of β2-AR prevents the increase in type 2 cytokine after *Clonorchis sinensis* infection. The experimental setup of the *in vivo* model used n = 4 mice in the control group and n=6 mice in the infected group. The mice were divided into control *Adrb2^+/^* ^+^, *Adrb2^−/−^* and infected *Adrb2^+/^* ^+^, *Adrb2^−/−^*. **(A)** The Masson’s staining of the liver tissue was carried out in the control and infected group. The infected group showed a significant accumulation of collagen fibers, as shown by the intense blue color; however, *Adrb2^−/−^* significantly decreased collagen fibers accumulation compared to *Adrb2^+/^* ^+^. **(B)** The alpha SMA expression in the liver of control and infected mice. The liver expression of alpha SMA in the control group was significantly lower than that observed in the infected group (*P < 0.001*). Also, the expression of alpha SMA in the infected group was significantly higher in infected *Adrb2^+/^* ^+^ than infected *Adrb2^−/−^* (*P < 0.001*). **(C)** The measurement of Hydroxyproline content showed an increase in the infected group than the control group, and the increase in the hydroxyproline content was higher in infected *Adrb2^+/^* ^+^ compared to infected *Adrb2^−/−^* (*P < 0.01*). **(D–F)** Measurement of the production of type 2 cytokines IL-4 and IL-13 and the relative mRNA expression of *TGF-beta* in liver tissue of control and infected mice. β2-AR-deficient mice prevented the increase in the production of IL-4, IL-13, and *tgf-beta* after *Clonorchis sinensis* infection. The infected *Adrb2^+/^* ^+^ showed a significant increase in the production of IL-4 (*P < 0.001*), and IL-13 (*P < 0.01*), and the increase in the mRNA expression of *tgf-beta* (*P < 0.001*), compared to the control group and infected *Adrb2^−/−^* group. Compared with indicated groups, **P < 0.01, ***P < 0.001.

Since *C. sinensis* can induce liver fibrosis in mammals, we firstly investigated whether the knocking out of β2-AR has effects on liver fibrosis caused by the worm or not. Masson’s staining, which allows the observation of ECM, is a stain used to detect liver fibrosis and liver cirrhosis. The results of this study showed that the control group (non-infected mice) did not show accumulation of collagen fibers, as shown by the lack of blue strips. In contrast, as shown in **Figure 3A**, the accumulation of collagen fibers represented by the blue color was significantly decreased in infected *Adrb2^−/−^* mice, compared with infected *Adrb2^+/+^* mice. Similarly, compared to the control group without infection, alpha-smooth-muscle actin (α-SMA), which is an indicator of fibroblast activity, increased in both *Adrb2^+/+^* and *Adrb2^−/−^* mice infected with *C. sinensis.* However, the β2-AR-deficient mice infected by *C. sinensis* showed a significant decrease in α-SMA expression compared to the *Adrb2^+/+^* infected group (**Figure 3B**, *P<*0.001). Besides, hydroxyproline content, which is a common marker of liver fibrosis, was much higher in the infected group compared to the non-infected control group. *C. sinensis* infection induced a significant increase in hydroxyproline content in *Adrb2^+/+^* mice, while the knocking out of β2-AR in mice (*Adrb2^−/−^* mice) significantly decreased hydroxyproline content (**Figure 3C**, *P*<0.01). These data together indicate that β2-AR deficiency abolished liver fibrosis caused by worm infection.

### Knocking Out of β2-AR Prevents the Increase in Type 2 Cytokines After *C. sinensis* Infection

A characteristic of helminth infection is the upregulation of type 2 immune response. Type 2 immune response characterized by type 2 cytokines plays an important role in liver fibrosis as IL-4, IL-13, and TGF-β1 produced by macrophages can activate HSCs leading to liver fibrosis (24). Since some studies have shown that *C. sinensis* infection leads to type 2 immune response in FVB mice (25), we decided to check whether β2-AR can regulate type 2 immune response during liver fluke infection or not. Not surprisingly, the results showed that *C. sinensis* infection increases type 2 cytokine production, especially IL-4 and IL-13, in infected *Adrb2^+/+^* compared to the non-infected *Adrb2^+/+^* (**Figures 3D, E**). Furthermore, our results revealed that *Adrb2^−/−^* mice infected with *C. sinensis* showed significantly lower production of type 2 cytokines, in particular, IL-4 (**Figure 3D**, *P<0.001*) and IL-13 (**Figure 3E**, *P*<0.01), compared to that observance in *Adrb2^+/+^* mice with worm infection. The level of *tgfb1* mRNA, which indicates type 2 immune response, was also significantly decreased in infected *Adrb2^−/−^* mice compared to *Adrb2^+/+^* mice (**Figure 3F**, *P*<0.01).

### *Adrb2^−/−^* Significantly Decreased Alternatively Activated Macrophages Infiltration in *C. sinensis* Infection

**Figure 4:**
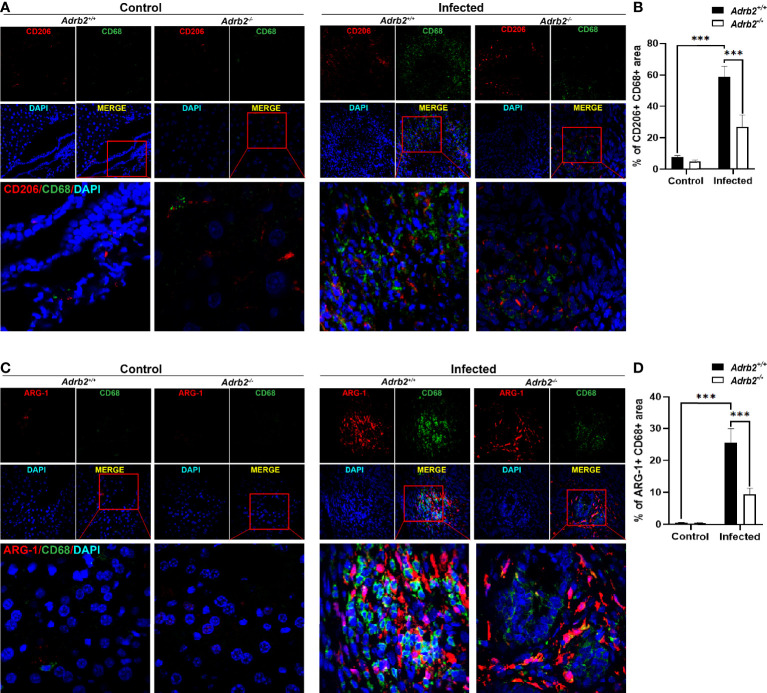
*Adrb2^−/−^* significantly decreased alternatively activated macrophages infiltration in *Clonorchis sinensis* infection. The experimental setup of the *in vivo* model used n = 4 mice in the control group and four mice in the infected group. The mice were divided into control *Adrb2^+/^* ^+^, *Adrb2^−/−^* and infected *Adrb2^+/^* ^+^, *Adrb2^−/−^*. **(A, B)** The immunofluorescence co-staining of CD68 and CD206 indicating the alternatively activated macrophages revealed that there was a strong infiltration of alternatively activated macrophages in infected mice than that observed in control mice; however, the knocking out of β2-AR induced a decrease in alternatively activated macrophages infiltration. **(C, D)** *In vivo* Immunofluorescence co-staining of CD68 and Arginase-1. The co-expression of CD68 and Arginase-1 indicating AAMs showed that there was a very low expression of CD68 and Arginase-1 in the liver of control mice. The infection of mice with *C. sinensis* resulted in a significant increase in the expression of arginase in the liver. Moreover, activated macrophages expressed arginase-1. However, *Adrb2^−/−^* mice infected with *C. sinensis* decreased the expression of positive CD68 and Arginase-1. Compared with indicated groups, ***P < 0.001.

AAMs are one of the most important sources of IL-4 and IL-13 during helminth infection, and liver fibrosis is associated with AAMs infiltration (26). In our present study, we further investigated the effects of the β2-AR on the AAMs in *C. sinensis*–infected mice. We co-stained CD68 with CD206 to define the AAMs in the liver section (27). Our data showed that the infiltrated macrophages highly expressed CD206 (**Figures 4A, B**, *P<0.001*). We also found that AAMs infiltration was higher in the infected group than that observed in the control group (**Figures 4A, B**, *P*<0.001). In addition, our data revealed that β2-AR-deficient mice significantly decreased the activation and infiltration of AAMs. We also found that arginase-1, which is highly expressed by AAMs, was increased in the liver of infected mice, but there was a very low expression of arginase-1 in the liver of control mice. Moreover, arginase-1 was highly expressed in infiltrated macrophages of infected *Adrb2^+/+^* mice, indicating that the infiltrated macrophages involved in *C. sinensis*–induced liver fibrosis are AAMs (**Figures 4C, D**, *P*<0.001).

### β2-AR Enhances Alternatively Activated Macrophages *In Vitro*

**Figure 5:**
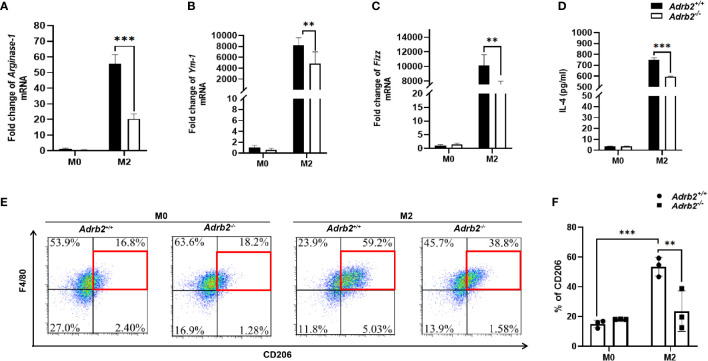
β2-AR enhances alternatively activated macrophages *in vitro*. The experimental setup of the *in vitro* model used bone marrow–derived macrophages (BMDM). BMDM was divided into two groups, M0 (unstimulated BMDM) and M2 or alternatively activated macrophages (IL-4 stimulated BMDM for 24 h). The concentration of recombinant IL-4 used to stimulate the BMDM was 20 ng/ml. Compared to the unstimulated macrophages, the IL-4-stimulated macrophages showed a significant increase in M2 markers. **(A–C)** The mRNA levels of *Arginase-1*, *Ym-1*, and *Fizz* were increased in alternatively activated macrophages. **(D)** ELISA’s result showed a significant increase in the production of IL-4 in alternatively activated macrophages. **(E, F)** The flow cytometry result showed a significant increase in the percentage of CD206 mannose receptors in alternatively activated macrophages. β2-AR-deficient alternatively activated macrophages decreased the M2 markers such as IL-4, Arginase-1, Ym-1, Fizz, and CD206 compared to *Adrb2^+/^* ^+^ alternatively activated macrophages. Compared with indicated groups, **P < 0.01, ***P < 0.001.

The previous results of this work have demonstrated that β2-AR-deficient (*Adrb2^−/−^*) mice show a decrease in liver fibrosis following *C. sinensis* infection, which may be associated with M2 macrophages. To investigate the mechanism by which β2-AR regulates AAMs, we isolated and cultured bone marrow–derived macrophages from *Adrb2^−/−^* mice and *Adrb2^+/+^* mice. In this study, we generated AAMs by IL-4 stimulation to highlight the role of β2-AR in the regulation of AAMs. Thus, we tested whether macrophages stimulated by IL-4 in this study express M2 markers. The expression of M2 marker genes such as *Arginase-1, Fizz-1, Ym-1* was significantly upregulated in IL-4-stimulated macrophages (**Figures 5A–C**). Similarly, the percentage of CD206, which is a surface marker of M2 macrophages, was also significantly upregulated (**Figures 5E, F**, *P*<0.001). Type 2 cytokine IL-4 was also significantly upregulated in M2 macrophages compared to M0 (unstimulated macrophages) (**Figure 5D**). These data suggest that we successfully induced AAMs with the stimulation of IL-4. We then asked whether β2-AR regulated the M2 macrophages response *in vitro* or not. M2 *Adrb2^−/−^* macrophages stimulated by IL-4 downregulated the expression of M2 markers such as *Arginase-1*, *Fizz*, and *Ym-1*, compared to M2 *Adrb2^+/+^* macrophages (**Figures 5A–C**). Also, the percentage of CD206 in β2-AR-deficient (*Adrb2^−/−^*) macrophages was significantly decreased compared to that of *Adrb2^+/+^* macrophages (**Figures 5E, F**, *P*<0.01). IL-4, which is one of the most important type 2 cytokines, was significantly upregulated in *Adrb2^+/+^* M2 macrophages compared to *Adrb2^−/−^* M2 macrophages (**Figure 5D**, *P*<0.001).

### β2-AR Controls M2 Macrophage *via* ERK/mTORC1 Signaling *In Vitro*

**Figure 6:**
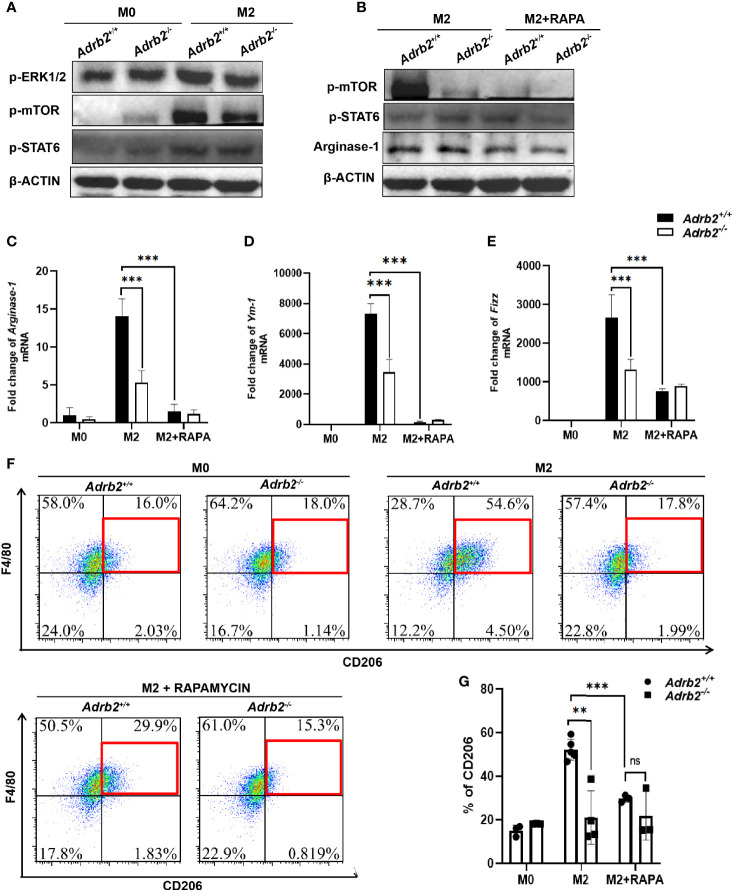
β2-AR controls the type 2 inflammatory response *in vitro* in ERK/mTORC1 signaling. The experimental setup of the *in vitro* model used bone marrow–derived macrophages (BMDM). BMDM was divided into three groups: M0 (unstimulated macrophages), M2 or alternatively activated macrophages (IL-4-stimulated macrophages for 24 h), and M2 + Rapamycin (100 ng/ml for 24 h). **(A)** The increase in M2 markers in M2 *Adrb2^+/^* ^+^ macrophages is associated with an increase in the phosphorylation of ERK1/2 and mTORC1. **(B)** The inhibition of mTOR by Rapamycin significantly decreases the phosphorylation of mTOR, resulting in the decrease of Arginase-1. **(C–E)** The inhibition of mTORC1 significantly decreases the mRNA level of M2 markers such as *Arginase-1* **(C)**, *Ym-1* **(D)**, and *Fizz* **(E)** in M2 *Adrb2^+/^* ^+^ macrophages. **(F, G)** The inhibition of mTORC1 also decreases the percentage of CD206 in M2 *Adrb2^+/^* ^+^ macrophages. Compared with indicated groups, **P < 0.01, ***P < 0.001; ns, not significant.

It has previously been demonstrated that mTOR plays an important role in the metabolic reprogramming of the M2 macrophages (17). To investigate the pathway by which β2-ARs regulate type 2 immune response in macrophages, we firstly investigated β2-AR-mediated canonical signaling. Our results revealed that M2 macrophages (IL-4-stimulated macrophages) did not result in a difference in PKA phosphorylation in *Adrb2^+/+^* macrophages and *Adrb2^−/−^* macrophages compared to M0 macrophages (unstimulated macrophages) (**Supplementary S1B**). More interestingly, no differences in the phosphorylation of AKT and PI3K, which are downstream of PKA and upstream of mTOR, were observed between M2 *Adrb2^+/+^* and M2 *Adrb2^−/−^* macrophages, indicating that canonical signaling was not involved in the control of inflammation by β2-AR (**Supplementary S1B**). Subsequently, we investigated whether non-canonical signaling through MAPK was involved in this process or not. Our results showed that IL-4 stimulation increased the phosphorylation of ERK1/2 in M2 *Adrb2^+/+^* macrophages compared to M2 *Adrb2^−/−^* macrophages (**Figure 6A**). The phosphorylation of ERK1/2 in M2 *Adrb2^+/+^* macrophages was associated with an increase in mTOR phosphorylation (**Figure 6A**), indicating that the control of inflammation by β2-AR occurred along the non-canonical signaling pathway. Moreover, M2 *Adrb2^−/−^* macrophages showed a significant decrease in the phosphorylation of mTOR compared to M2 *Adrb2^+/+^* macrophages. In addition, compared to M2 macrophages, M0 (unstimulated macrophages) did not induce phosphorylation of mTOR, indicating that mTOR was essential for IL-4 to induce AAMs.

To investigate the mechanism by which β2-AR controls type 2 inflammation in macrophages by mTOR, we checked whether the inhibition of mTORC1 by Rapamycin can decrease the M2 markers. Compared to the M2 control macrophage, the inhibition of mTORC1 in M2 macrophage significantly decreased the phosphorylation of mTOR in *Adrb2^+/+^* (**Figure 6B**). Moreover, Rapamycin completely abolished the increase of M2 macrophage markers in *Adrb2^+/+^* (**Figures 6C–G**). Compared to M2 *Adrb2^−/−^* macrophages, the decrease of M2 markers in M2 *Adrb2^−/−^* macrophages inhibited with Rapamycin was not significant (**Figures 6C–G**). The above results indicated that the inhibition of mTORC1 by Rapamycin inhibited mTOR phosphorylation and the decrease in type 2 inflammatory response in macrophages. However, inhibition of mTORC1 by Rapamycin does not induce any change in Stat6 phosphorylation, indicating that the signaling by which β2-ARs regulate M2 macrophages is through mTORC1 and independent of Stat6 (**Figure 6B**).

## Discussion

The autonomic nervous system regulates the activity of immune cells through a set of signals. Macrophages possess receptors that allow them to respond to signals from the autonomic nervous system. Alternatively activated macrophages (M2) are important in the process of tissue repair and the maintenance of homeostasis; however, little is known about the regulatory roles of β2-AR in the activation of M2. In the present study, compared with *Adrb2^+/+^* mice, we found that *Adrb2* ^−/−^ mice showed alleviated hepatobiliary injuries and fibrosis after *C. sinensis* infection, which is associated with reduction of M2 activation and its relative cytokines (IL-4 and IL-13). *In vitro*, we also found that β2-AR orchestrated the activation of M2 associated with mTOR. Our present study provides a good understanding of the mechanisms by which β2-AR is involved in the regulation of M2 macrophages.

FVB mice by their ability to elicit predominant type 2 immune response during helminth infection are a widely used model for the study of type 2 immune response (25, 28). We found that the infection of mice with *C. sinensis* results in strong cholangiocyte proliferation (**Figure 2B**). Cholangiocyte activation has been reported to play an important role in Kupffer cell activation and hepatic stellate cells as well, and the infiltration of macrophages promotes cholangiocyte proliferation (29, 30). In general, cholangiocytes proliferate in response to an injury. The control of cholangiocyte proliferation is an important element in liver fibrosis because the activation of cholangiocytes leads to the activation of hepatic stellate cells, which are the main source of tumor growth factor-beta 1 (TGF-β1).

In the liver, resident macrophages (Kupffer cells) play an important role in maintaining homeostasis, orchestrating tissue remodeling in ontogenesis, and regulating metabolic functions (31), while infiltrated bone marrow–derived macrophages play a role in the fighting of the parasite (31). During the parasite infection, an elevated type 2 immune response can limit the excessive inflammatory reaction followed by fibrosis and ultimately cirrhosis. It has previously been reported that during *C. sinensis* infection, macrophages are the main source of type 2 immune response (32). The results of this study revealed that the infection of mice with *C. sinensis* results in significant macrophages activation and infiltration. In the liver, activated and infiltrated macrophages play a very important role in the expulsion of the parasite; however, the excessive inflammatory reaction can lead to liver damage (31). Moreover, there was significant infiltration of AAMs, which are the principal contributor to liver fibrosis. Here we have shown that the β2-AR-deficient mice significantly show a decrease in the activation and infiltration of macrophages as well as the prevention of liver fibrosis.

AAMs promote fibrosis by the upregulation of type 2 cytokines such as IL-4, IL-13, and TGF-β, leading to activation of hepatic stellate cells (33–35). The role of β2-AR in the regulation of inflammatory response has been the subject of several works. In classically activated macrophages, the activation of β2-AR prevents the increase of type 1 immune response by the increase in the cAMP/PKA level (36). In our present study, we found that the activation of β2-AR in AAMs promotes type 2 immune response. Our results are consistent with previous studies that have shown that an increase in the inflammatory response in macrophages is associated with the activation of ERK1/2 rather than cAMP/PKA (37). M2 macrophages are characterized by a significant increase in M2 markers such as *Arginase-1*, *Fizz-1*, *Ym-1*, CD206, and can be induced by type 2 cytokines such as IL-4 and IL-13 (4, 5, 38). The mechanism by which AAMs are fine-regulated during their differentiation has not yet been investigated. Although it is known that the autonomic nervous system regulates the activity of immune cells, no investigation has been carried out into the mechanism by which the autonomic nervous system regulates the activity of AAMs. In this study, we did not observe the production of IL-13 in M2 macrophages stimulated by IL-4. This might be because we used IL-4 to induce the differentiation of macrophages mediated by IL-4Rα but not IL-13. β2-AR-deficient AAMs showed a significant reduction of the M2 markers compared to β2-AR wild-type macrophages. The increase in M2 markers may be associated with an increase in metabolism as shown by the increase in mTOR in *Adrb2^+/+^* compared to *Adrb2^−/−^* macrophages. Previous studies have suggested that IL-4 acts synergistically with M-CSF to induce M2 macrophages through PI3K/AKT/mTORC2 and Stat6 signaling (17). It has previously been demonstrated that the differentiation of macrophages from M0 to M2 macrophage requires mTORC2, but not mTORC1 signaling since non-selective mTORC2 inhibitor “Torin,” but not “Rapamycin,” a selective mTORC1 inhibitor, inhibits Stat6, induces M2 macrophages differentiation (17). In our study, M2 *Adrb2^+/+^* macrophages and M2 *Adrb2^−/−^* macrophages show an increase in AAMs marker compared to M0 macrophages (in which there were not any M2 markers). However, the M2 *Adrb2^+/+^* macrophages show a significant upregulation of AAMs marker compared to M2 *Adrb2^−/−^* macrophages. In addition, we did not observe any changes in AKT and PI3K phosphorylation, which are upstream of mTORC2, between *Adrb2^+/+^* and *Adrb2^−/−^* macrophages, which might suggest that mTORC2 is not involved in the regulation of inflammatory response by β2-AR. Moreover, the inhibition of mTORC1 significantly decreases M2 activation in *Adrb2^+/+^* compared to that observed in M2 *Adrb2^+/+^* without mTOR inhibition, indicating that mTORC1 is implicated in β2-AR-induced alternatively activated macrophages regulation. This result showed that the inhibition of mTORC1 does not inhibit the differentiation of macrophages from M0 to M2, but decreases M2 macrophages response.

The control of inflammatory response by β2-AR involves the canonical and non-canonical signaling pathways. In our present study, AAMs enhanced the M2 phenotype by the activation of ERK1/2-mTORC1 signaling, indicating that the regulation of AAMs by β2-AR occurred along with the non-canonical signaling. ERK1/2-mTORC1 has also been shown to induce epithelial-mesenchymal transition and fibrosis in retinal pigment epithelial cells (39). Also, stimulation of macrophages with LPS has been shown to enhance type 1 cytokines in ERK1/2 signaling (40). However, further investigations are needed to highlight the downstream as well as the mechanism by which mTORC1 controls AAMs.

## Conclusion

This study provides a better understanding of the mechanisms by which the β2-AR regulates type 2 immune response through the ERK/mTORC1 signaling pathway in macrophages and their roles in liver fibrosis. We found that β2-AR-deficient mice showed a significant decrease in the activation and infiltration of AAMs, which is associated with the reduction of liver fibrosis after *C. sinensis*. Our results also showed that the control of AAMs occurs along with the non-canonical ERK1/2 and mTORC1 signaling. All these data together suggest that β2-AR regulates AAMs in the ERK/mTORC1 signaling pathway during liver fibrosis induced by helminth infection.

## Data Availability Statement

The raw data supporting the conclusions of this article will be made available by the authors, without undue reservation.

## Ethics Statement

The animal study was reviewed and approved by the Animal Experiments of Xuzhou Medical University and the National Guide for the Care and Use of Laboratory Animals (201701w007).

## Author Contributions

CY and K-YZ conceived and designed the experiments. SK and BZ performed the majority of experiments. Q-YZ, NX, JL, J-XL, ML, Z-YL, J-LW, YS, SG, QY, X-YL,Y-HX, J-XC, R-XT, and HS contributed to the acquisition of data. BT and GA revised the English. SK, CY and BZ wrote the paper. All authors contributed to the article and approved the submitted version.

## Funding

This study was supported by the National Natural Science Foundation of China (Grant Nos: 82172297 to K-YZ and 81702027 to QY), Natural Science Foundation of Jiangsu Province of China (Grant No. BK20211346 to CY and BK20201011 to BZ), China Postdoctoral Science Foundation (Grant No. 2018M640525 to CY), Qian Lan project of Jiangsu Province (to CY), Jiangsu Planned Projects for Postdoctoral Research Funds (No. 2018K053B to CY and No. RC7062005 to BZ), the starting grants for young scientist of Xuzhou Medical University (No. D2019040), Priority Academic Program Development of Jiangsu Higher Education Institutions of China (K-YZ) and Graduate research project of Jiangsu Province (Grant No. KYCX20-2468 to JL). The funders had no role in study design, data collection, and analysis, decision to publish, or preparation of the manuscript.

## Conflict of Interest

The authors declare that the research was conducted in the absence of any commercial or financial relationships that could be construed as a potential conflict of interest.

## Publisher’s Note

All claims expressed in this article are solely those of the authors and do not necessarily represent those of their affiliated organizations, or those of the publisher, the editors and the reviewers. Any product that may be evaluated in this article, or claim that may be made by its manufacturer, is not guaranteed or endorsed by the publisher.

## Supplementary Material

Supplementary Figure 1
**(A)** HE staining of the bile duct of normal and *Clonorchis sinensis–*infected mice. *C. sinensis* induces the dilatation of the bile duct in infected mice. Moreover, the dilation of the bile duct is more severe in infected *Adrb2^+/^* ^+^ than that observed in infected *Adrb2^−/−^*. **(B)** Western blot of the *in vitro* experiment on BMDM. The stimulation of macrophages by IL-4 to induce AAMs does not induce a significant change in the phosphorylation of PKA and AKT compared with non-stimulated macrophages. **(C)** The experimental procedure used for the *in vitro* experiment on macrophages.
