# Supplementary figures and images for: β2-Adrenergic Receptor Enhances the Alternatively Activated Macrophages and Promotes Biliary Injuries Caused by Helminth Infection
Source: Front Immunol. 2021 Oct 18;12:754208. doi: 10.3389/fimmu.2021.754208 (PMC8558246; doi:10.3389/fimmu.2021.754208)

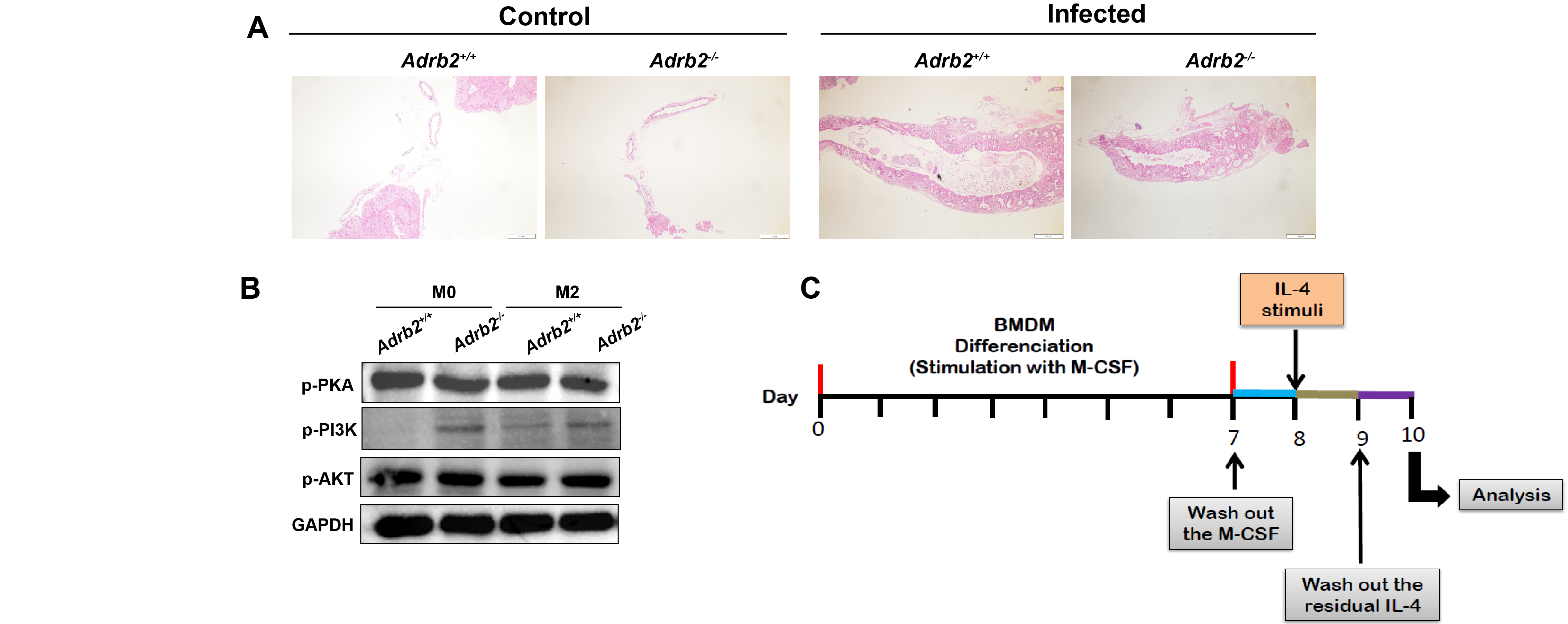

Supplement: Supplementary Figure 1 — (A) HE staining of the bile duct of normal and Clonorchis sinensis–infected mice. C. sinensis induces the dilatation of the bile duct in infected mice. Moreover, the dilation of the bile duct is more severe in infected Adrb2+/ + than that observed in infected Adrb2−/− . (B) Western blot of the in vitro experiment on BMDM. The stimulation of macrophages by IL-4 to induce AAMs does not induce a significant change in the phosphorylation of PKA and AKT compared with non-stimulated macrophages. (C) The experimental procedure used for the in vitro experiment on macrophages. [file Image_1.jpeg]
